# Application of an Artificial Neural Network in the Diagnosis of Chronic Lymphocytic Leukemia

**DOI:** 10.7759/cureus.4004

**Published:** 2019-02-04

**Authors:** Fateme Shaabanpour Aghamaleki, Behrouz Mollashahi, Mokhtar Nosrati, Afshin Moradi, Mojgan Sheikhpour, Abolfazl Movafagh

**Affiliations:** 1 Genetics, Shahid Beheshti University of Medical Sciences, Tehran, IRN; 2 Genetics, University of Isfahan, Isfahan, IRN; 3 Pathology, Shahid Beheshti University of Medical Science, Tehran, IRN; 4 Genetics, Pasteur Institute of Iran, Tehran, IRN

**Keywords:** artificial neural network, biomarkers, diagnosis, chronic lymphocytic leukemia

## Abstract

Introduction

Chronic lymphocytic leukemia (CLL) is one of the most common types of leukemia, and the early diagnosis of patients coincides with their proper treatment and survival. If patients are diagnosed late or proper treatment is not applied, it may lead to harmful results. Several methods could be used for the diagnosis of leukemia; some of these include complete blood count (CBC), immunophenotyping, lymph node biopsy, chest X-ray, computerized tomography (CT) scan, and ultrasound. Most of these methods are time-consuming and an application of more than one method will result as intended. This acknowledgment stresses the necessity of rapid and proper diagnosis for leukemia based on clinical and medical findings, inasmuch as it was decided to apply the artificial neural network (ANN) in order to identify a molecular biomarker for rapid leukemia diagnosis from blood samples and evaluate its potential for the detection of cancer.

Materials & methods

The independent sample t-test was applied with the Statistical Package for the Social Sciences (SPSS; IBM Corp, Armonk, NY, US) software on the microarray gene expression data of Gene Expression Omnibus (GEO) datasets (GSE22529); 12 genes that had shown the highest differences (among parameters whose p-value was less than 0.01) were selected for further ANN analysis. The selected genes of 53 patients were applied to the training network algorithm, with a learning rate of 0.1.

Results

The results showed a high accuracy of the relationship between the output of the trained network and the test data. The area under the receiver operating characteristic (ROC) curve was 0.991, which provides proof of the precision and the relationship with identifying Gelsolin as a potential biomarker for this research.

Conclusions

With these results, it was concluded that the training process of the ANN could be applied to rapid CLL diagnosis and finding a potential biomarker. Besides, it is suggested that this method could be performed to diagnose other forms of cancer in order to get a rapid and reliable outcome.

## Introduction

Chronic lymphocytic leukemia (CLL) is a type of blood and bone marrow cancer in which uncontrolled and abnormal growth of lymphocytic cells occur. CLL is also characterized by clonal proliferation and progressive accumulation of B-cell lymphocytes that typically express cluster of differentiation 19+ (CD19+), CD5+, and CD23+. CLL progresses slowly and each year, there are more cases added to its list. CLL happens quite frequently in adults in contrast to acute leukemia, which is more frequent in children [1-3]. The survival rate in CLL cancer is significantly higher than in any other types of cancer, as around 83% of the patients show a five-year survival rate, which means 83% of patients with CLL are living at least five years after the diagnosis is made. The etiology of CLL is still elusive, however, it is most likely that genetics and environmental factors have an important effect on its occurrence [4-5]. In the karyotype experiments, del13q14, trisomy 12, del11q22-q23, and del17p13 were associated with CLL [6]. Several other molecular pathologies were found in CLL, such as the overexpression of the unmutated immunoglobulin heavy chain variable region (IGHV) genes, zeta-chain-associated protein kinase 70 (ZAP-70), CD38 proteins, and mutations in the NOTCH1, splicing factor 3b subunit 1 (SF3B1), and baculoviral IAP repeat-containing 3 (BIRC3) genes. In addition, mutations in tumor suppressor genes, including tumor protein P53 (TP53) and ataxia-telangiectasia mutated (ATM), have been associated with degrees of resistance to common chemotherapeutic agents. Micro-ribonucleic acid (RNA) expression alterations and aberrant methylation patterns in genes, which are specifically deregulated in the CLL, including the B-cell lymphoma 2 (BCL-2), T-cell leukemia/lymphoma 1 (TCL1), and ZAP-70 genes, have also been encountered and linked to the distinct clinical parameters. The clinical manifestations of the diagnosis are extremely diverse [7-10]. Approximately 60% of the patients are asymptomatic, and the patient may be suspected of the disease after a routine blood test. When symptomatic, patients show unclear symptoms of fatigue or weakness. CLL is usually diagnosed with blood tests because the cancerous cells are found in the blood. A bone marrow biopsy is usually not needed to diagnose the CLL, but it may be done before the beginning of the treatment. Recently, molecular and cellular markers have helped with the prediction and diagnosis of CLL in patients. Therefore, the identification of key molecules in CLL could be important and vital in order to find a more effective diagnosis of CLL [2,11-12].

The gene-expression profiling using cDNA microarrays gives us the ability to simultaneously analyze multiple markers, which helps us categorize cancers into subgroups. Although, many statistical techniques to analyze the gene-expression data exist, none of them have been precisely tested for their ability to accurately distinguish different types of cancers and to further categorize them based on their diagnostic methods [13-14].

The artificial neural networks (ANNs) method is a computer-based algorithm that is modeled on the structure and behavior of neurons in the human brain and could be trained to recognize and categorize complex patterns. The pattern recognition is achieved by adjusting the parameters of the ANN by a process of error minimization through learning from experiences. The parameters could be calibrated using any type of input data, such as gene-expression levels generated by cDNA microarrays, and the output could be grouped into any given number of categories [15-16]. The ANN has been recently applied to the clinical cases, such as the diagnosis of myocardial infarction and arrhythmias from their respective electrocardiograms, and the interpretations of their radiographs and magnetic resonance imaging (MRI). The ANN correctly classifies all the samples and identifies the genes that are most related to the classifications [17-18].

In summary, the purpose of this study was to develop a method for classifying cancers to specific diagnostic categories based on their gene expression signatures using ANNs [19-20]. This study demonstrated the potential applications of these methods for CLL diagnosis and the identification of candidate targets (or genes) for diagnosis and therapy.

## Materials and methods

Microarray gene expression profile

The gene expression profile of CLL patients was extracted from the Gene Expression Omnibus (GEO) database under the accession number of GEO series 22529 (GSE22529), which was based on GEO platform 96 (GPL96) ((HG-U133A) Affymetrix Human Genome U133A array) and deposited by Jelinek D, Kay N. This data was submitted on June 23, 2010, and updated on August 10, 2018. The gene expression profile was generated from peripheral blood samples (n=104). This data contained the gene expression profile of 104 serum samples, including 81 patients and 23 healthy controls. In this study, the gene expression of 53 samples, including 11 healthy controls and 42 patients with CLL were selected for further analysis and validation of the ANN model.

Selection of genes by highest score

Initially, the expression levels of 22285 genes were ranked. Then, after a primary statistical analysis, the two-tailed student t-test was used to determine the statistical significance for the difference between the two groups of CLL patients and healthy individuals. The genes were selected based on the significance of their differences and were used as the inputs for the ANN analysis. Statistical analysis was carried out by the Statistical Package for the Social Sciences (SPSS; IBM Corp, Armonk, NY, US) 18 software.

Artificial neural network (ANN)

A multilayer perceptron ANN model with three layers was performed by RapidMiner software (RapidMiner, Inc., MA, US). The three layers consisted of an input layer, a hidden layer, and an output layer. The input layer contained 12 neurons corresponding to 12 input features; the hidden layer applied learning algorithm to the input features. Finally, the output layer had only one neuron, representing two possible diagnosis states of cancerous or noncancerous. The values of the output layer were 0 and 1, which were categorized as the cancerous and healthy control, respectively. Initially, the ANN was trained using the 12 genes with the highest scores as the input. Finally, to evaluate the ANN model, the area under the receiver operating characteristic (AUROC), the classification accuracy, and an index about reliability were calculated. The ROC curve is the plot of sensitivity (true positive rate) against 1-specificity (false positive rate), which was created by the GenEx version 6 software (MultiD Analyses, Göteborg, Sweden) for all of the 12 inputs to recognize their differences separately and to select the best gene as a diagnostic biomarker. In this study, The Decision Tree and the Support Vector Machine (SVM) algorithms were also used to examine the ANN algorithm.

## Results

The first step of the process was selecting the significant genes. According to the t-test, the top 12 genes were identified as significant (p-value<0.001); they had the most differences between the two groups of healthy individuals and patients. These genes were selected from our dataset for further analyses (Table 1).

**Table 1:** A list of the twelve selected genes based on the t-test result. These twelve genes were selected based on their lowest p-value. The probe ID of the genes in the microarray and their names and gene symbols were identified.

Probe ID	Gene symbol	Species	Gene name
200666_s_at	DNAJB1	Homo sapiens	DnaJ heat shock protein family (Hsp40) member B1
200627_at	PTGES3	Homo sapiens	Prostaglandin E Synthase 3
200664_s_at	DNAJB1	Homo sapiens	DnaJ heat shock protein family (Hsp40) member B1
200701_at	NPC2	Homo sapiens	NPC intracellular cholesterol transporter 2
200675_at	CD81	Homo sapiens	CD81 molecule
200028_s_at	STARD7	Homo sapiens	StAR related lipid transfer domain 7
200634_at	PFN1	Homo sapiens	Profilin 1
200709_at	FKBP1A	Homo sapiens	FK506 binding protein 1A
200022_at	RPL18	Homo sapiens	Ribosomal Protein L18
200696_s_at	GSN	Homo sapiens	Gelsolin
200657_at	SLC25A5	Homo sapiens	Solute Carrier Family 25 Member 5
200650_s_at	LDHA	Homo sapiens	Lactate Dehydrogenase A

Then, to compare the cancerous patients with healthy groups, 12 features or genes were considered. Three algorithms were initially used to compare these two groups: the ANN, The Support Vector Machine, and The Decision Tree. Then, the algorithm with a better outcome was selected for the purpose of diagnosis. The results of this test are presented in Table 2. According to this analysis, it could be understood that the algorithm of the ANN could better distinguish the differences between the two groups with an accuracy of 99% (AUC=0.991, CA=0.969, F1=0.969); therefore, for the main analysis, this algorithm had been selected [21].

**Table 2:** The results of three algorithms for the twelve genes in two groups of patients and healthy. AUC = Area under curve in the ROC analysis, CA = Classification accuracy, F1 = An index of reliability

Method	AUC	CA	F1	Precision	Recall
SVM	0.985	0.952	0.953	0.955	0.952
Random Forest	0.969	0.936	0.936	0.936	0.936
Neural network	0.991	0.969	0.969	0.970	0.969

In the next step, the training process of the created neural network was performed for the purpose of diagnosis. According to Table 3, it is clear that the algorithm of the neural network has been able to correctly distinguish the two groups of patients from healthy with 98% accuracy based on the expression of those 12 genes. The area under the ROC curve was measured to estimate the diagnostic performance of the ANN. Table 3 shows the results of cross-validation using the ANN algorithm.

**Table 3:** The results of the neural network algorithm for the twelve genes between the two groups. The results of the neural network algorithm for the 12 genes between the two groups. This algorithm was applied for the 12 selected genes, to understand the ANN value in classification. ANN = Artificial neural network, AUC = Area under curve in the ROC analysis, CA = Classification accuracy, F1 = An index of reliability

Method	AUC	CA	F1	Precision	Recall
Neural network	0.991	0.981	0.980	0.981	0.981

**Figure 1:**
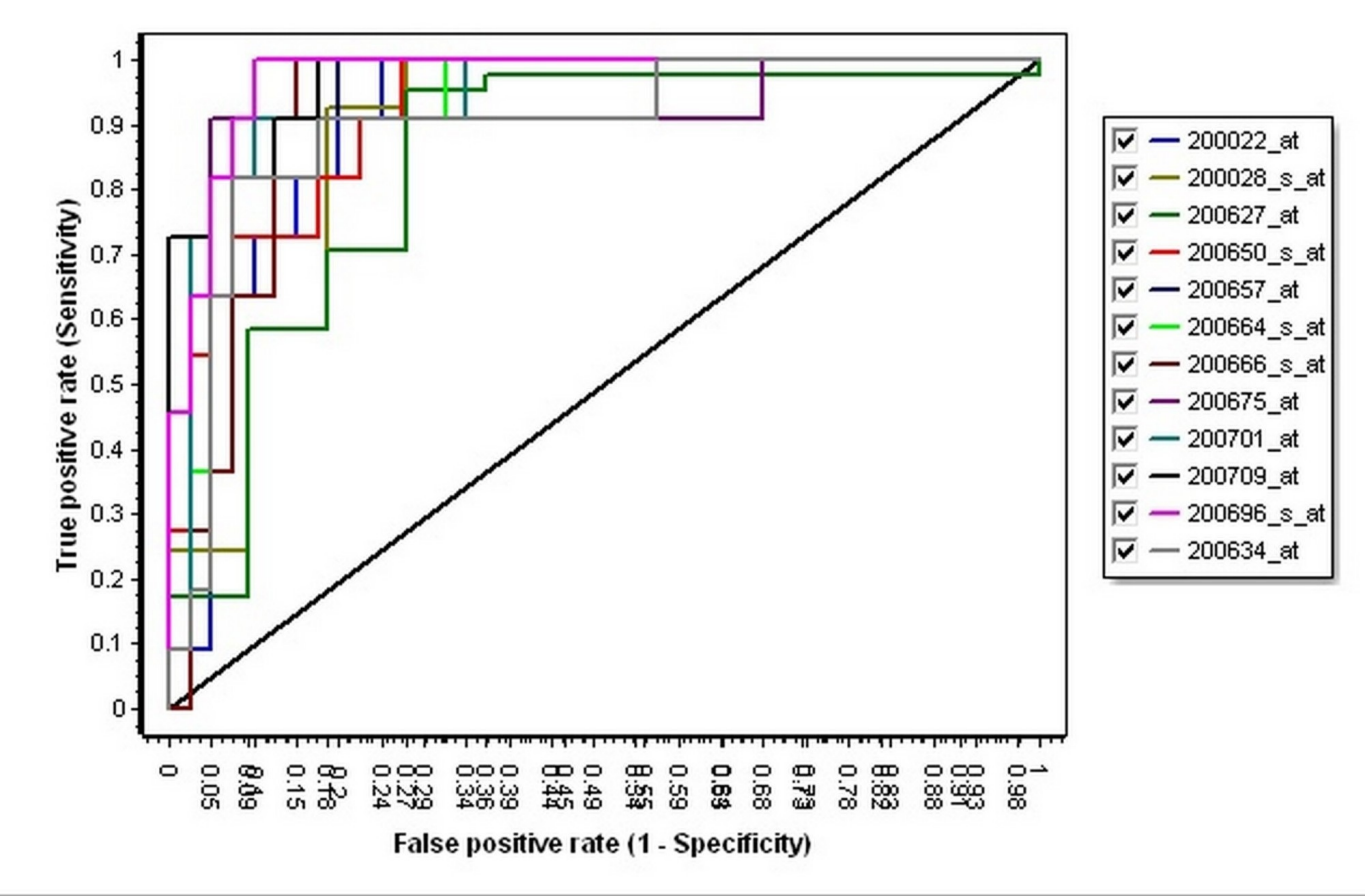
The receiver operating characteristic curve of the twelve genes The plot of each gene is indicated with a special color. The horizontal axis is defined as specificity and the vertical axis is defined as sensitivity.

**Figure 2:**
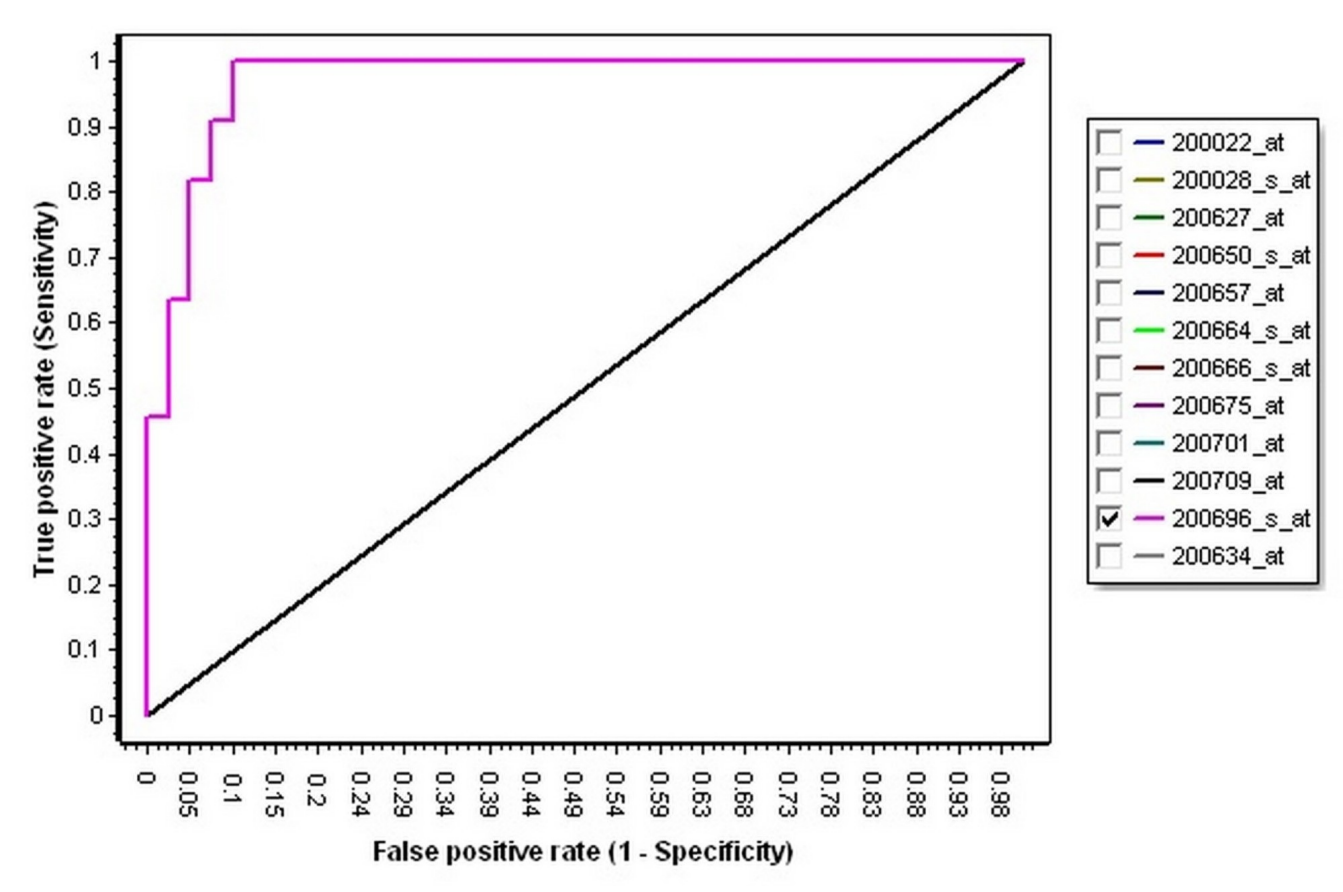
The receiver operating characteristic curve of Gelsolin. The ROC curve of GSN with the highest AUC; the AUROC curve is 0.991, which is higher than the AUROC of the other genes. ROC = receiver operating characteristic, GSN = Gelsolin, AUC = Area under curve; AUROC: Area under the receiver operating characteristic

Then, in order to identify the best gene in the 12 selected genes as a diagnostic biomarker, the ROC curve analysis and plots for all of the 12 genes were calculated and determined (Table 4, Figure 1). According to Table 4, the Gelsolin (GSN) with AUC = 0.971, specificity = 0.902, and sensitivity = 1, could be a better diagnostic biomarker for the diagnosis of the CLL (Figure 2).

**Table 4:** Results of receiver operating characteristic curve analysis of the twelve genes. Results of the ROC curve analysis of the 12 genes were indicated and according to this result, GSN has higher AUC; therefore, it can diagnose CLL samples as compared to healthy samples. AUC = Area under the curve in the ROC analysis, ROC = receiver operating characteristic, GSN = Gelsolin, CLL = Chronic lymphocytic leukemia

Probe ID	Gene symbol	Specificity	Sensitivity	AUC
200022_at	RPL18	0.756097561	1	0.911308204
200028_s_at	STARD7	0.818181818	0.926829268	0.88691796
200627_at	PTGES3	0.727272727	0.951219512	0.840354767
200650_s_at	LDHA	0.731707317	1	0.922394678
200657_at	SLC25A5	0.804878049	1	0.953436807
200664_s_at	DNAJB1	0.902439024	0.909090909	0.931263858
200666_s_at	DNAJB1	0.853658537	1	0.922394678
200675_at	CD81	0.951219512	0.909090909	0.911308204
200701_at	NPC2	0.902439024	0.909090909	0.940133038
200709_at	FKBP1A	0.829268293	1	0.968957871
200696_s_at	GSN	0.902439024	1	0.971175166
200634_at	PFN1	0.926829268	0.818181818	0.89578714

## Discussion

The diagnosis of the CLL in the early stages may lead to an increase in the survival rate and might demonstrate better therapeutic results. While taking a biopsy from the bone marrow still remains the golden standard for screening CLL, the approach has several shortcomings including its invasiveness and the patient’s discomfort. Contrarily, the non-invasive tests, such as the blood test, has low sensitivity and specificity [22-23]. The previous studies were used to indicate that the genes were potential candidates for the diagnosis of cancer in its early stages. We used the ANN analysis to provide more improvements and advancements in order to pinpoint cancer more accurately, in its early stages, and to further evaluate this computer technique potential in the assay of detecting cancer since the examination of the expression levels of a panel of genes could be used to classify patients into cancerous and healthy individuals.

Recently, the application of the ANN has found its way into medical fields. Although the ANN training algorithms vary, they share one basic function: all networks accept a set of inputs and, based on their hidden layer algorithm, generate corresponding outputs. The ANN is particularly practical for medical cases without a linear solution. In the presented study, an ANN model provided good predictive accuracy as a diagnostic biomarker for a precise classification. It was hypothesized that not only could this technique increase the accuracy of the CLL diagnostic tests, but it could also be applied to several other types of cancer diagnoses. In this paper, we made the effort to obtain sample data from the GEO database in order to classify them as cancerous and healthy individuals. Twelve genes, as listed in Table 1, were shown to be appropriate for the accurate diagnosis of CLL using the ANN algorithm. The accuracy of the detection of cancer by the assembled ANN was analyzed by the ROC analysis. The outputs of the trained ANN for testing data were used to plot the ROC curve, and the area under the ROC curve was 0.991. These results demonstrated the high performance of the ANN training in the diagnosis of CLL according to its gene expression and the good learning process of the ANN, therefore, a panel of genes could be used for the ANN algorithm to detect CLL.

Based on the ANN results and ROC curve, it could be stated that GSN has a potential diagnostic biomarker value. GSN plays many roles in various types of cancer. Gelsolin is a ubiquitous actin filament-severing protein, one of the most important members of the actin-severing superfamily, and plays a crucial role in the regulation of actin filament assembly and disassembly. Additionally, it has an important responsibility in many other cellular properties, such as carcinogenesis phenotypes, epithelial-mesenchymal transition (EMT), motility, apoptosis, proliferation, and differentiation [24-26]. GSN overexpression has been seen in many cancers, including breast cancer, oral carcinoma cells, colorectal cancer, ovarian cancer, and leukemia [27-28].

## Conclusions

In conclusion, by collaborating the t-test and the ANN, it is possible to identify a minimum and an optimum number of gene biomarkers for the classification of healthy and CLL individuals. Based on the gene expression values, a trained ANN model accurately classified the sample data into the cancerous and non-cancerous categories. As a result, it was shown that the learning technique of the ANN could accurately differentiate cancerous samples from the non-cancerous. It could also choose the potential biomarker gene in a more time-efficient manner, which could result in better diagnosis and better treatment.
